# Perception of the McGurk effect in people with one eye depends on whether the eye is removed during infancy or adulthood

**DOI:** 10.3389/fnins.2023.1217831

**Published:** 2023-10-13

**Authors:** Stefania S. Moro, Faizaan A. Qureshi, Jennifer K. E. Steeves

**Affiliations:** ^1^Department of Psychology and Centre for Vision Research, York University, Toronto, ON, Canada; ^2^The Hospital for Sick Children, Toronto, ON, Canada

**Keywords:** monocular enucleation, audiovisual processing, McGurk effect, face processing, voice processing, multisensory, visual development

## Abstract

**Background:**

The visual system is not fully mature at birth and continues to develop throughout infancy until it reaches adult levels through late childhood and adolescence. Disruption of vision during this postnatal period and prior to visual maturation results in deficits of visual processing and in turn may affect the development of complementary senses. Studying people who have had one eye surgically removed during early postnatal development is a useful model for understanding timelines of sensory development and the role of binocularity in visual system maturation. Adaptive auditory and audiovisual plasticity following the loss of one eye early in life has been observed for both low-and high-level visual stimuli. Notably, people who have had one eye removed early in life perceive the McGurk effect much less than binocular controls.

**Methods:**

The current study investigates whether multisensory compensatory mechanisms are also present in people who had one eye removed late in life, after postnatal visual system maturation, by measuring whether they perceive the McGurk effect compared to binocular controls and people who have had one eye removed early in life.

**Results:**

People who had one eye removed late in life perceived the McGurk effect similar to binocular viewing controls, unlike those who had one eye removed early in life.

**Conclusion:**

This suggests differences in multisensory compensatory mechanisms based on age at surgical eye removal. These results indicate that cross-modal adaptations for the loss of binocularity may be dependent on plasticity levels during cortical development.

## 1. Introduction

The visual system is not fully mature at birth and continues to develop throughout infancy until it reaches adult levels in late childhood and adolescence (see Daw, 2014 for a review). Multiple critical periods exist in postnatal visual development where different visual functions are maturing along with their underlying neural substrates. During a critical period, the nervous system is vulnerable to environmental stimuli where, if appropriate stimuli are not provided, the development of that function may be hindered or eliminated. There are three types of critical periods present during postnatal visual development: The critical period of development, where environmental experience has an impact on a function while it is rapidly developing; the critical period for disruption, where a postnatal experience has an adverse effect on the development of a function; and the critical period of recovery, where a disrupted function can be recovered (Daw, 2014). Critical periods for different visual functions such as acuity or motion perception can emerge at different times (Daw, 2014). For example, visual acuity does not fully develop to adult sensitivity levels until the age of 4–6 years (Mayer and Dobson, 1982; Ellemberg et al., 1999), directional motion sensitivity may achieve adult sensitivity levels between the age of 3 years and adolescence (Ellemberg et al., 2002; Parrish et al., 2005; Hadad et al., 2011), and specialized visual processing such as facial recognition peaks around age 30 years (Germaine et al., 2011).

A unique model for examining the consequences of the loss of binocularity is unilateral eye enucleation the surgical removal of one eye (see Steeves et al., 2008, for a review). Unlike other forms of monocular visual deprivation such as cataract or strabismus that leave abnormal visual input, removing the eye completely denies all forms of visual input to the brain from that eye (Steeves et al., 2008). Early monocular enucleation (prior to 5 years of age) is a particularly useful model of study since the visual system has not been exposed to abnormal visual input from the removed eye. Losing one eye early in life, during postnatal visual system maturation, has been shown to lead to both enhanced and reduced visual function. These enhancements and reductions in performance depend on whether one is measuring visual spatial ability or visual motion processing and oculomotor systems (reviewed in Steeves et al. (2008) and Kelly et al. (2013)). People with one eye demonstrate superior contrast sensitivity at 2, 4, and 8 cycles/degree compared to control participants viewing with their non-dominant eye patched (Nicholas et al., 1996). Furthermore, people who lost their eye before 2 years of age have better contrast sensitivity at 4 cycles/degree compared to those who lost their eye at a later age, and moreover, compared to binocular viewing (BV) controls (Nicholas et al., 1996). These results indicate a developmental relationship between age at enucleation and contrast sensitivity, where earlier enucleation leads to larger improvement in contrast sensitivity with the remaining eye and likely facilitates cortical remapping to underlie this ability.

More recently, studies have focused on whether early monocular eye enucleation in humans results in adaptations across other senses similar to those with early complete blindness (i.e., Lessard et al., 1998). Auditory localization is consistently more accurate in all locations (i.e., within 78 degrees to the left or right of straight ahead) except for the extreme periphery in people with one eye compared to control participants who were binocular viewing, eye-patched, or had both eyes closed. Moreover, people with one eye also demonstrate improved monaural (one ear) sound localization and did not show the typical tendency to mislocate sounds towards the ‘straight ahead’ as did controls (Hoover et al., 2012). Adaptive audiovisual plasticity following the loss of one eye early in life has also been observed for both low-and high-level audiovisual stimuli. People with early eye enucleation do not show the typical pattern of visual dominance when asked to categorize rapidly presented audiovisual targets, suggesting enhanced weighting is applied to the auditory component of a bimodal stimulus (Moro and Steeves, 2012, 2013). Audiovisual processing differences vary depending on the nature of the stimuli presented where low-level flash and beep stimuli are identified by people who had one eye removed early in life similar to binocular viewing controls but with longer response latencies (Moro et al., 2014; Moro and Steeves, 2018a). People who had one eye removed early in life do not retain the same visual benefit from observing faces as binocular viewing participants for face-voice identity recognition despite performing with response latencies similar to binocular viewing controls (Moro et al., 2018). Furthermore, people who have had one eye removed early in life have a reduced susceptibility for audiovisual illusions, namely the double flash illusion where participants perceive a single flash of light as two flashes when presented with concurrent multiple beeps, and the McGurk Effect, an audiovisual illusion where a new syllable is perceived when visual lip movements do not match the corresponding auditory sound (McGurk and MacDonald, 1976; Moro and Steeves, 2018b,c) despite performing with response latencies similar to binocular viewing controls.

The accurate perception of speech is highly influenced by audiovisual integration. Auditory speech information is greatly enhanced by the presence of visual lip movements, especially under noisy conditions (Szycik et al., 2012). The McGurk illusion has become a popular tool for studying the mechanisms underlying multisensory integration, despite having substantial inter-subject variability (Beauchamp et al., 2010; Alsius et al., 2017). Three factors might contribute to individual differences in the perception of the McGurk illusion: (1) superior sensitivity to detecting audiovisual correspondences, where the auditory and visual sensory signals would not erroneously be attributed as belonging to the same event; (2) higher/lower weighting of the visual or auditory cues, where the higher weighted modality will more greatly contribute to the perception of the event; (3) an inefficient combination of the two cues, where poorer integration will contribute to the perception of individual auditory and visual events that are not fused into a single event (Alsius et al., 2017). Neural substrates have also been implicated in accounting for individual differences in the perception of the McGurk effect where functional magnetic resonance imaging has shown that greater activation of the left STS was correlated with greater perception of the McGurk effect (Nath and Beauchamp, 2012).

People who have had one eye removed early in life perceive the McGurk effect less often than binocular and eye-patched viewing controls (Moro and Steeves, 2018c) but with similar response latencies. Clinically, evidence of a decreased McGurk effect in people with amblyopia, a neural developmental vision disorder, has been observed (Narinesingh et al., 2014). The decreased perception of the McGurk effect in amblyopia persists during binocular and fellow eye viewing conditions indicating that the underlying causes are neural and associated with more complex sensory processes that are not specific to visual acuity (Narinesingh et al., 2014). Much of the previous research has focused on investigating the impact of multisensory compensatory mechanisms in people who had one eye removed early in life (prior to 2 years of age). Understanding whether multisensory compensatory mechanisms are also present in people who had one eye removed later in life and whether there is a difference in compensatory mechanisms based on time since eye removal (number of years since enucleation) or age of eye removal (age eye was removed), or experience with binocularity is important for understanding the mechanisms of sensory plasticity. The current study is the first to investigate how people who have had an eye removed late in life, after visual system maturation, perceive the McGurk effect compared to people who have had one eye removed early in life, during postnatal visual system maturation, and binocular viewing controls. Given that there are different critical periods for the development of different visual functions (Mayer and Dobson, 1982; Ellemberg et al., 1999, 2002; Parrish et al., 2005; Germaine et al., 2011; Hadad et al., 2011; Daw, 2014) and that children have been found to be less susceptible to the McGurk (Tremblay et al., 2007; Narinesingh et al., 2015) it is possible that individuals who have one eye removed later in life will not exhibit the previously observed differences in audiovisual processing that have been documented in people who had one eye removed at a young age during visual system maturation. It is possible that a lack of modulation of the McGurk effect in late eye enucleated individuals could be due to changes to the visual system after the developmental critical period of multisensory processing and perhaps also after the critical periods for disruption or recovery.

## 2. Materials and methods

### 2.1. Participants

#### 2.1.1. People with late monocular enucleation

Eight adult participants who had undergone monocular eye enucleation (L-ME) participated in this study (mean age = 50 years, SD = 13 years). All L-ME participants with one eye had been unilaterally eye enucleated (3 right eye removed) due to various reasons including traumatic injury, cancer, or infection. Age at enucleation ranged from 5 years to 55 years (mean age at enucleation = 23 years, SD = 15 years).

#### 2.1.2. People with early monocular enucleation

Eight adult participants who had undergone monocular eye enucleation (E-ME) at The Hospital for Sick Children in Toronto participated in this study (mean age = 34 years, SD = 13 months). All E-ME participants with one eye had been unilaterally eye enucleated (6 right eye removed) due to retinoblastoma, a rare childhood cancer of the retina. Age at enucleation ranged from 4 months to 66 months (mean age at enucleation = 24 months, SD = 19 months). All E-ME data were previously reported in Moro and Steeves (2018c) to test a different hypothesis.

#### 2.1.3. Binocular viewing control participants

Thirty binocularly intact controls with a mean age of 31 years (SD = 13 years) were tested viewing stimuli with both eyes.

An *a priori* power analysis was conducted using G*Power version 3.1 (Faul et al., 2007) for sample size estimation for a repeated-measures, within factors ANOVA. With a significance criterion of *α* = 0.05 and power = 0.95, the minimum sample size needed for a medium (0.25) effect size is *N* = 36 participants.

All participants (L-ME, E-ME, and BV) reported normal hearing and normal or corrected-to-normal visual acuity and were instructed to wear optical correction if needed. All participants gave informed consent prior to their inclusion in the study, which was approved by York University Office of Research Ethics. All L-ME and 10 of the BV participants completed this study online. There was no difference in performance between the online and in-person platforms and as a result the data were collapsed across platforms (see Supplementary material for more detail). E-ME data were collected in-person and were previously reported in a study conducted by Moro and Steeves (2018c).

### 2.2. Stimuli

**Figure 1:**
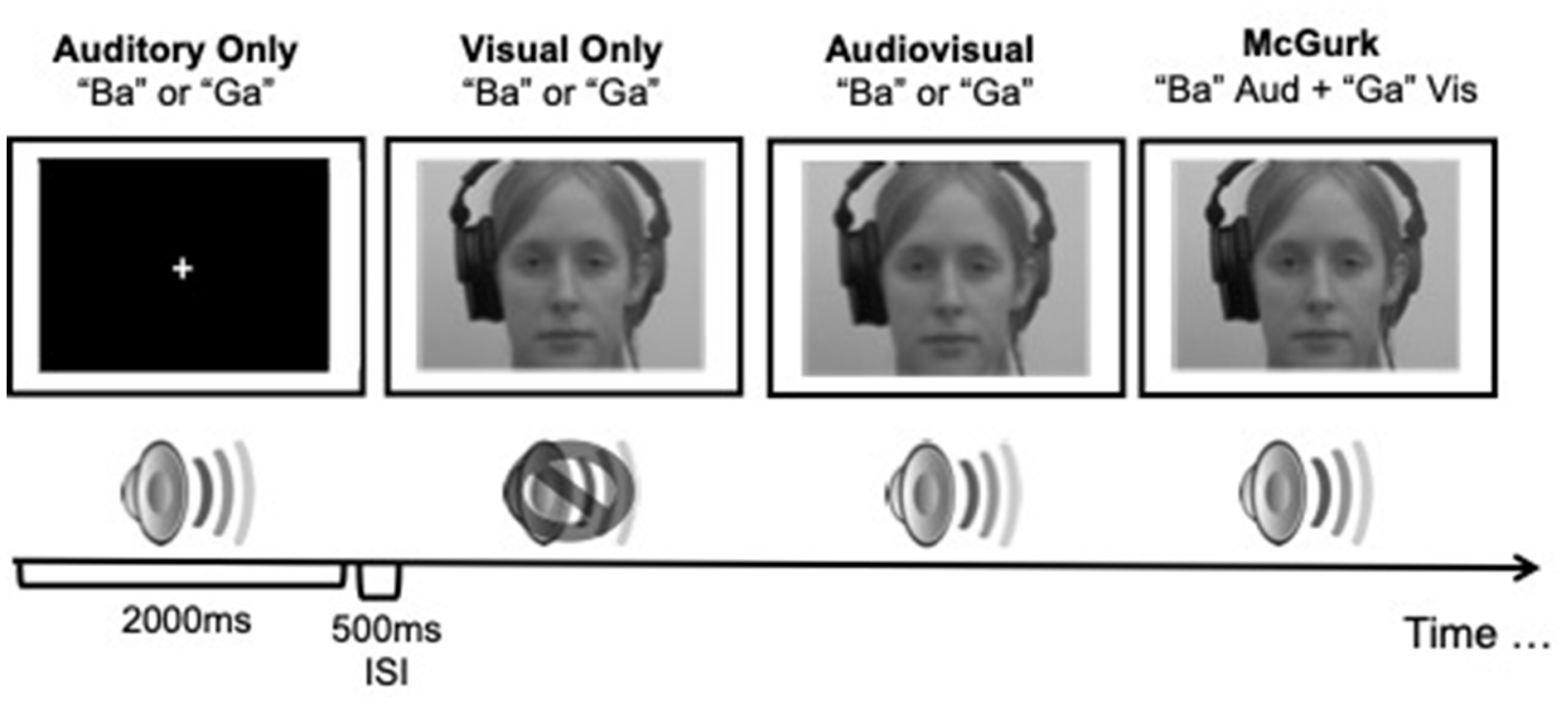
A schematic illustration of the presentation of stimuli used in the McGurk effect study. Visual stimuli were presented to participants in colour. Adapted from Moro and Steeves (2018c).

All stimuli were identical to those used in Moro and Steeves (2018c). Briefly, visual stimuli consisted of two 2 s videos of a female speaker mouthing the syllables “Ba” and “Ga,” with each presentation containing the entire articulation of the syllable similar to those used by Quinto et al. (2010). Auditory stimuli consisted of 2 s audio clips of the female speaker from the videos saying the syllables “Ba” and “Ga.” Audiovisual stimuli consisted of two 2 s videos of the female speaker saying the syllables “Ba” and “Ga,” paired with the corresponding video, respectively. McGurk illusory stimuli consisted of video footage of the female speaker mouthing the “Ga” syllable but paired with the auditory sound clip of the female speaker saying “Ba” (Figure 1). The McGurk effect is observed by measuring the participant’s syllable perception (“Ba,” “Ga,” or the illusory perception of “Da”). There were a total of 4 conditions (auditory only, visual only, audiovisual, and illusory McGurk) and participants viewed 40 repetitions per condition with a 500 ms interstimulus interval consisting of silence and a blank screen for a total of 160 trials. L-ME and BV participants completed the study online with stimuli programmed using PsychoPy, an open-source psychophysics software (Pierce et al., 2019), presented on Pavlovia, an online stimulus presentation software platform (Open Science Tools). E-ME participants completed the study in the laboratory with identical stimuli presented using SuperLab stimulus presentation software (Cedrus Inc.).

### 2.3. Procedure

All participants were instructed to respond as quickly as possible to unimodal visual, unimodal auditory, or bimodal stimuli. Online participants were prompted to set their computer volume to a comfortable listening level prior to beginning the study by adjusting the sound of a tone played through Pavlovia (Open Science Tools). Online participants were also prompted to calibrate their monitor to ensure consistent presentation of the visual stimuli by manually adjusting the size of a rectangle using their keyboard to the size of a credit card (Morys-Carter, 2021). Online participants were instructed to indicate using their keyboard what they perceived the woman in the video said (“Ba,” “Ga,” or “Da”) by indicating their response on one of the three designated keys. Participants who completed the study in-person sat at a distance of 60 cm from a 21.5″ computer screen in a dimly lit testing room. Auditory stimuli were presented to the participants using *Sony Studio Monitor Series* noise cancelling headphones placed over their ears with the volume regulated to a comfortable hearing level by participants. All participants were given written instructions and a short practice session consisting of 10 trials for familiarization with the task. The full experiment took about 7 min to complete. In-person E-ME data were collected according to the methods previously reported in Moro and Steeves (2018c).

## 3. Results

All statistical analyses were completed using jamovi v.2.3.21 (jamovi project, 2022), Prism v.9.3.1 (GraphPad Software, Inc., 2021), and R (Pinheiro, et al., 2023).

### 3.1. Comparing online and in-person platforms

To determine whether there were differences in accuracy, reaction time, or perception of the McGurk effect between online and in-person platforms for binocular viewing control participants we conducted a number of comparisons using repeated measures analysis of variance (ANOVA). Overall, there was no difference between the two platforms and as a result the two platforms were collapsed to yield one BV control data set (see Supplementary material for more details).

### 3.2. Accuracy

**Figure 2:**
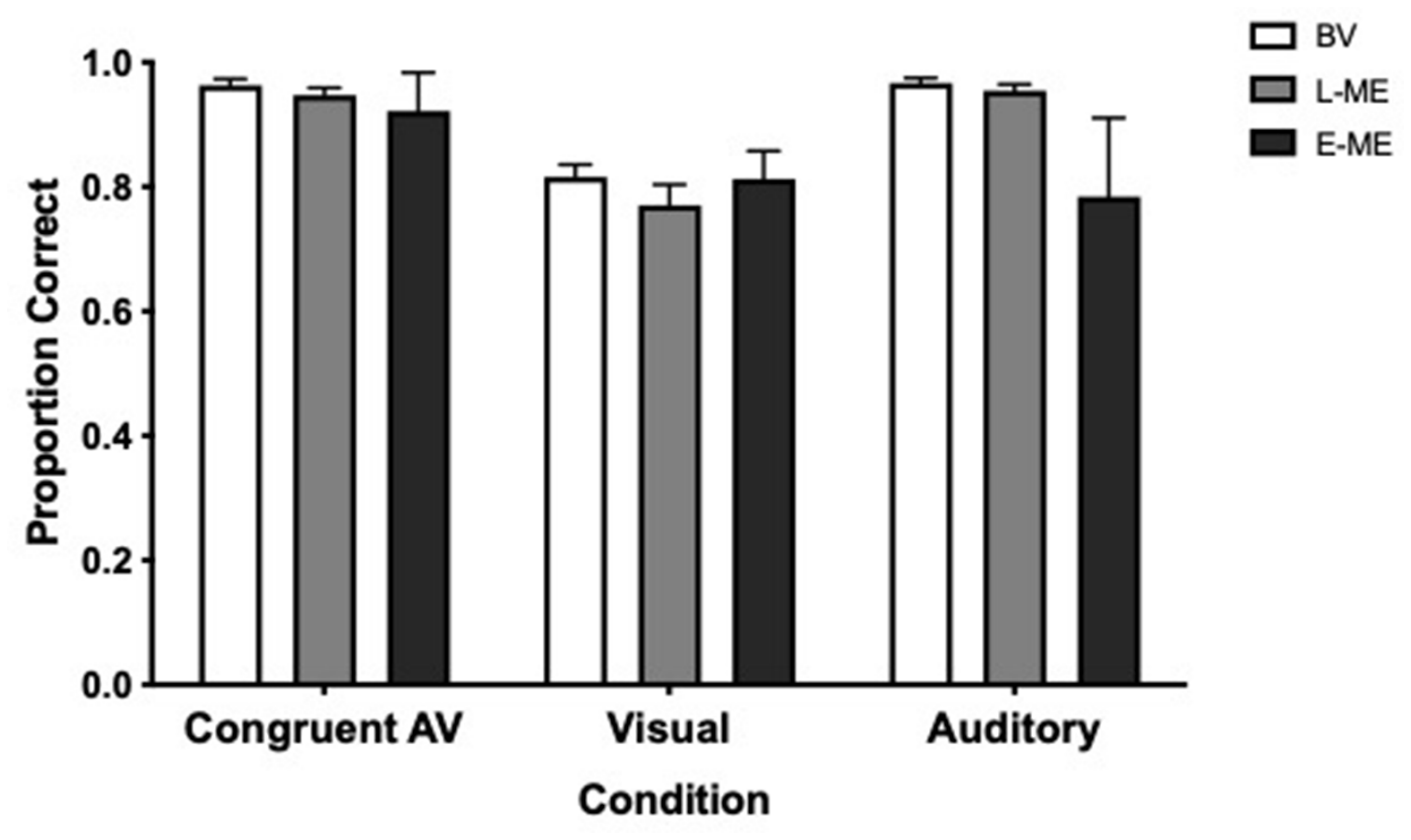
Accuracy for each condition for the BV (white), L-ME (grey), and E-ME (black) groups. There was no difference in accuracy between the groups. Error bars represent the standard error of the mean (SEM).

To determine whether there was a difference in auditory, visual, congruent audiovisual perception performance with respect to Accuracy between participant groups a Greenhouse–Geisser corrected, 3 × 3 repeated measures analysis of variance (ANOVA) comparing Participant Group (BV vs. L-ME vs. E-ME) and Condition (auditory only, visual only, congruent audiovisual) was conducted. There was no significant interaction, *F*(2.79, 60.03) = 2.65, *p* = 0.061, *ŋ*_p_^2^ = 0.110. There was a significant main effect of condition, *F*(1.40, 60.03) = 23.56, *p* < 0.001, *ŋ*_p_^2^ = 0.354 and a significant main effect of participant group, *F*(2, 43) = 3.22, *p* = 0.050, *ŋ*_p_^2^ = 0.130. Tukey corrected post-hoc tests indicated that there was no difference between groups at each of the different stimulus conditions. Figure 2 plots the Accuracy for each for the BV, L-ME, and E-ME groups.

### 3.3. McGurk effect

**Figure 3:**
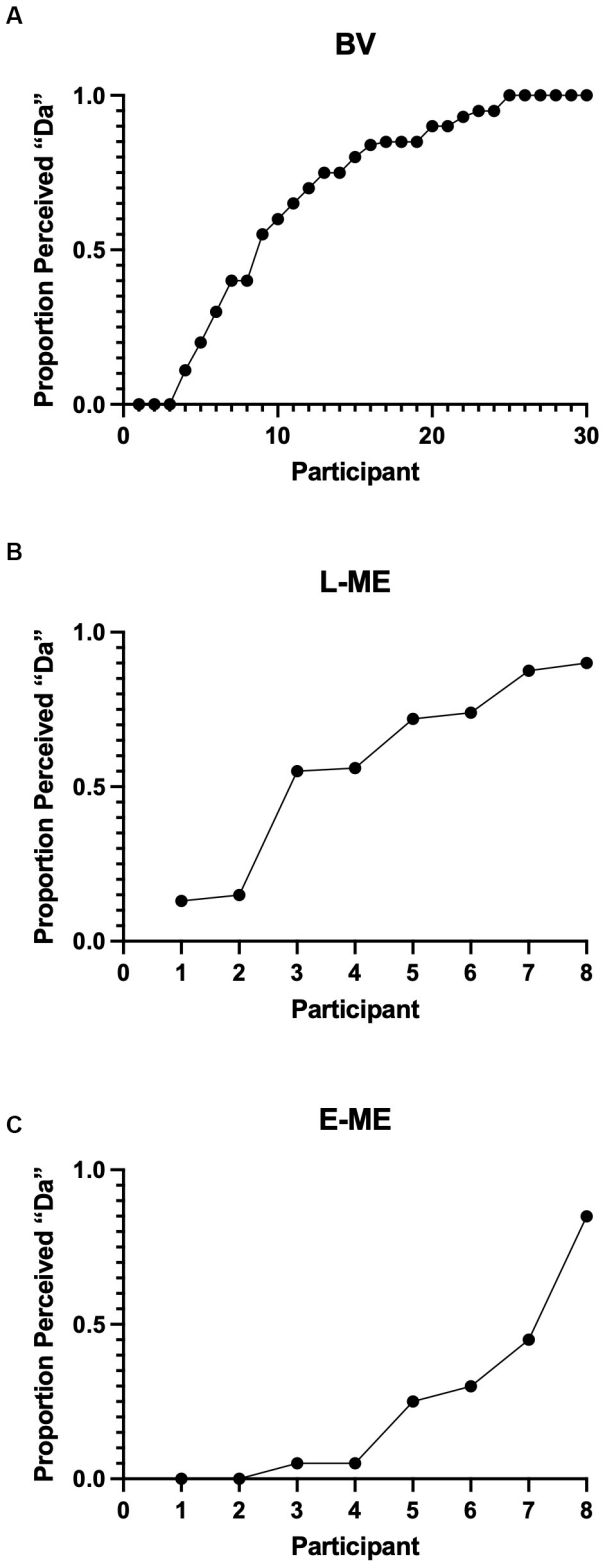
**(A)** Perception of the McGurk effect for the BV **(A)**, L-ME **(B)**, and E-ME **(C)** groups. Mean frequencies of McGurk responses for each participant (represented by each circle) ordered by increasing frequency. The BV group demonstrated increased perception of the illusory “Da” compared to the perception of “Ba” and to the perception of “Ga.” The L-ME group showed increased perception of “Da” compared to the perception of “Ga.” The E-ME group did not show increased perception of “Da” compared to either “Ga” or “Ba”.

To determine whether there was a difference in perception of the McGurk Effect between participant groups a logistic regression, using a hierarchical model, comparing the probability of a “Da” response with a “Non-Da” response was conducted. In order to account for individual variability, the participant factor (SD = 2.428) was used as a random effect with random slopes and random intercepts. Results indicated a significant difference between groups: *X*^2^ (2, *N* = 46) = 9.449, *p* = 0.008. Bonferroni corrected, likelihood ratio chi-square pairwise comparisons indicate that the participants with early eye enucleation have a lower probability of selecting “Da” compared to the binocular viewing controls (*p* = 0.002). Aligned with the previous study by Moro and Steeves (2018c), there was no evidence of a difference in the probability for selecting “Da” for participants with late monocular enucleation compared to binocular viewing controls (*p* = 0.236) and participants with early monocular enucleation compared to late monocular enucleation (*p* = 0.126).” Figure 3 plots the perception of the McGurk effect for the individual participants of the BV, L-ME, and E-ME groups (see Table 1).

**Table 1:** Group comparison for the probability of selecting “Da” for participants with late monocular enucleation (L-ME), early monocular enucleation (E-ME), and binocular viewing controls (BV).

	Estimate	Standard error	*p*-value
BV vs. E-ME	−3.156	1.043	0.002
BV vs. L-ME	−1.192	1.006	0.236
E-ME vs. L-ME	−1.965	1.283	0.126

### 3.4. Correlation of visual experience with perception of the McGurk effect

**Figure 4:**
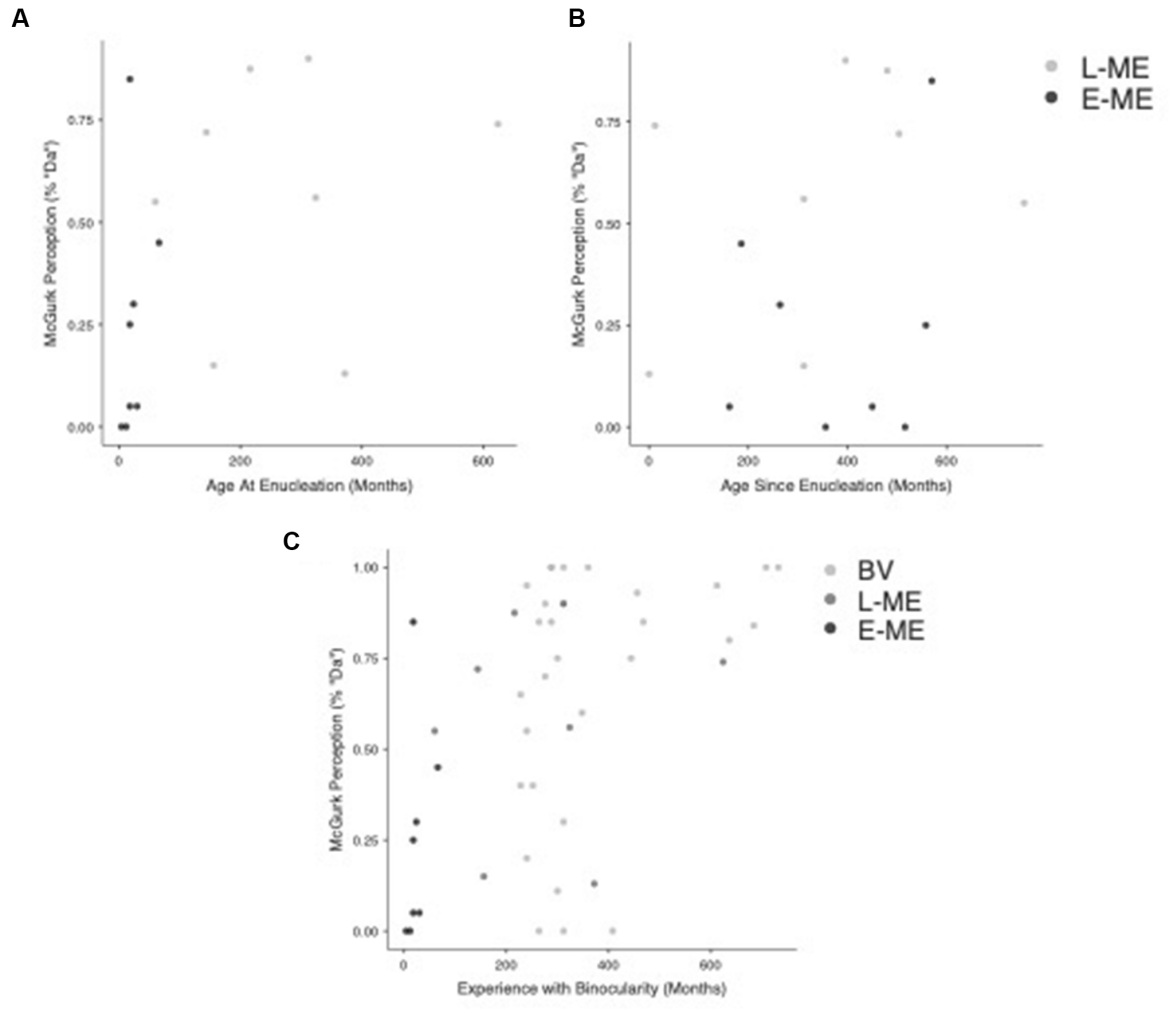
Correlation of visual experience with perception of the McGurk effect. **(A)** Plots the age at enucleation compared to perception of the McGurk effect for the L-ME (dark grey) and E-ME (black) groups. **(B)** Plots the age since enucleation compared to the perception of the McGurk effect for the L-ME (dark grey) and E-ME (black) groups. **(C)** Plots the relationship between experience with binocularity and the perception of the McGurk Effect for the BV (light grey), L-ME (dark grey), and E-ME (black) groups.

We investigated the relationship between age at enucleation (age in months that eye was removed) and time since enucleation (time in months since eye was removed) to the perception of the McGurk effect for both monocular enucleation groups (E-ME and L-ME). A Pearson correlation comparing age since enucleation and the perception of the McGurk effect, *r*(14) = 0.198, *p* = 0.462 was not significant. Figure 4A visualizes the relationship between age at enucleation and the perception of the McGurk effect for the L-ME and E-ME groups. A Pearson correlation comparing age at enucleation and the perception of the McGurk effect, *r*(14) = 0.444, *p* = 0.085 was not significant. Figure 4B visualizes the relationship between age since enucleation and the perception of the McGurk effect for the L-ME and E-ME groups.

We also investigated the relationship between experience of binocularity (the number of months lived with binocularity) compared to the perception of the McGurk effect for each group (BV, L-ME, and E-ME). A Pearson correlation comparing the experience of binocularity and the perception of the McGurk effect, *r*(43) = 0.491, *p* < 0.001 showed a strong positive correlation. Figure 4C Plots the relationship between experience with binocularity and the perception of the McGurk Effect for the BV, L-ME, and E-ME groups.

## 4. General discussion

The current study investigated how people with one eye removed late in life perceive the McGurk effect compared to people with one eye removed early in life and binocular viewing controls. Overall, people with one eye removed late in life perceived the McGurk effect, similar to binocular viewing participants, whereas people who had an eye removed early in life perceived the McGurk effect significantly less frequently. Furthermore, the length of time a participant experienced binocular vision was positively correlated with the perception of the McGurk effect. Therefore, increased experience with binocular vision led to a greater experience of the McGurk effect. These results suggest that disruption during visual development contributes to changes in the perception of audiovisual illusions, such as the McGurk effect. The resultant changes in the perception of the McGurk effect might be attributed to the three factors outlined by Alsius et al. (2017) such as (1) superior sensitivity to detecting audiovisual correspondences; (2) higher/lower weighting of the visual or auditory cues; (3) an inefficient combination of the two cues.

It has been shown that removing an eye early in life leads to altered audiovisual processing, such as reduced susceptibility to audiovisual illusions, namely the double flash illusion and the McGurk Effect (Moro and Steeves, 2018b,c). Additionally, people who have had an eye removed early in life do not display the typical pattern of visual dominance compared to controls when viewing line drawings paired with common sounds (Colavita effect: Moro and Steeves, 2012, 2013). An emerging pattern exists where people who have had an eye removed early in life have decreased susceptibility to audiovisual illusions with normal response latencies. Based on these findings, it is possible that the removal of one eye early in life might alter the ability to process sensory information, perhaps through higher/lower weighting of the visual or auditory cues, in order to achieve similar audiovisual integration performance compared to controls. Within visual processing, people who lose their eye before 2 years of age have better contrast sensitivity at 4 cycles/degree compared to those who lose their eye later, and moreover, compared to binocular viewing controls (Nicholas et al., 1996). This shows adaptive plasticity within the visual system based on interruption of early visual development. The current results indicate that differences in audiovisual processing might also be impacted by developmental experience with binocularity. When taken in context with the previous visual and audiovisual findings there is the possibility of a developmental relationship where earlier enucleation leads to larger improvement in contrast sensitivity with the remaining eye and likely facilitates cortical remapping to underlie this ability.

Age and context may impact sensory modality dominance (Diaconescu et al., 2013; Nava and Pavani, 2013; Robinson et al., 2016; Barnhart et al., 2018; Hirst et al., 2018b). A developmental trend exists where, compared to children, adults demonstrate stronger influence of unimodal visual stimuli when measuring audiovisual behaviour (Nava and Pavani, 2013; Wille and Ebersbach, 2016; Hirst et al., 2018a). Children are also more susceptible to illusions, such as the double flash illusion, where auditory information modulates visual perception (Innes-Brown et al., 2011) and less susceptible to the McGurk effect where they are less heavily influenced by incongruent visual information (Tremblay et al., 2007; Narinesingh et al., 2015). These findings indicate a developmental component associated with audiovisual integration that may be dependent on the development of sensory dominance throughout life, progressing from auditory dominance in childhood towards visual dominance in adulthood (Hirst et al., 2018a,b). It is likely that a disruption in early childhood, such as the reduction of 50% of the visual input to the brain *via* unilateral eye enucleation, will interfere with the development of audiovisual integration, accounting for the difference in perception of the McGurk effect in participants who had one eye removed early in life compared to late in life.

Children who have experienced early visual impairments are less susceptible to the McGurk effect (Narinesingh et al., 2014, 2015) whereas those with early hearing loss are more susceptible to the McGurk effect (Schorr et al., 2005). In children with amblyopia, this finding persists even when examined across the developmental trajectory where both children and adults who were diagnosed during childhood demonstrate reduced susceptibility to the McGurk effect (Narinesingh et al., 2015). Delayed development of multisensory integration processes through potential altered sensory weighting may account for the difference in McGurk illusory perception in people who had an eye removed early in life compared to those who have had an eye removed late in life. Early removal of an eye, before the completion of the critical period of visual development, may impact the overall weighting of unimodal sensory components therefore impacting the perception of the McGurk illusion in our patient group similar to what is observed in children. These results are also consistent with our previous findings where people who had their eye removed early in life do not display the typical pattern of visual dominance in the Colavita effect (Moro and Steeves, 2012, 2013) and do not retain the same visual benefit from face-voice identity recognition (Moro et al., 2018) compared to controls.

Limitations of the current study include sample size and variability across McGurk stimuli. Our current study compared 8 participants in each patient group to a group of 30 control participants. Despite our total sample size exceeding that predicted by our power analysis, future large-scale investigations across a larger group of people who have had an eye removed both early and late in life would strengthen the results of the current study. Additionally, variability across McGurk stimuli has also been found (Mallick et al., 2015). In the present study, only one syllable combination of the McGurk stimulus was used: Auditory “Ba” combined with Visual “Ga” with a female speaker. Furthermore, results indicated a decrease in accuracy for detecting the visual-only stimuli compared to the auditory-only and audiovisual stimuli. Despite each group achieving approximately 80% accuracy in this condition, there is likely increased difficulty associated with lipreading, specifically for the “Ga” syllable, that has more discrete lip movements. These findings further contribute to the variability across McGurk stimuli. Results should be interpreted with caution as they may not generalize to other McGurk stimuli. Future studies investigating audiovisual behaviour, as well as cortical structure and function in people who have had one eye removed early compared to late in life will help illustrate the impact of disrupted visual development on multisensory processing. Previous studies have indicated sub-cortical and cortical differences in brain structure and function in people who have had one eye removed early in life compared to binocular controls. These results include significant degeneration of the anterior visual system, including decreased optic chiasm volume and width (Kelly et al., 2014), decrease in lateral geniculate nucleus (LGN) volume (although the LGN volume contralateral to the remaining eye is less reduced) compared to binocular viewing controls (Kelly et al., 2014), larger left compared to right medial geniculate body (MGB) regardless of which eye was removed (Moro et al., 2015), and increased surface area and gyrification in visual, auditory and multisensory cortices compared to binocular viewing controls (Kelly et al., 2015). Taken together, there is moderate alterations of auditory and visual cortical and subcortical structures following early eye enucleation. Comparing neuroimaging findings across individuals who had one eye removed before visual system maturation, with those who had one eye removed later in life, after visual system maturation, and who have subsequently lived for a shorter time since enucleation, will help us to better understand the role of the critical periods of development, disruption, and recovery.

In conclusion, people who had one eye removed late in life perceive the McGurk effect more similarly to binocular controls unlike people who had one eye removed early in life. These results contribute to the likelihood that accommodations are present for audiovisual processing in people with one eye but that they may be dependent on the developmental period in which the eye was removed. Furthermore, it is possible that the developmental period when the eye was removed contributes to altered weightings of the auditory and visual modality impacting the proficiency of audiovisual integration. Further investigations aimed at measuring the developmental impact on the presence of sensory accommodations may facilitate classifying the adaptive compensatory mechanisms developed to account for the loss of half of the visual input to the brain.

## Data availability statement

The raw data supporting the conclusions of this article will be made available by the authors, without undue reservation.

## Ethics statement

The studies involving humans were approved by York University Ethics Review Board. The studies were conducted in accordance with the local legislation and institutional requirements. The participants provided their written informed consent to participate in this study.

## Author contributions

SSM and JKES: conceptualization and methodology. SSM and FAQ: software programming and investigation. SSM: formal analysis, writing—original draft, visualization, and project administration. JKES: validation, resources, and writing—review and editing, supervision, and funding acquisition. FAQ: writing—editing. All authors contributed to the article and approved the submitted version.
