# Supplementary figures and images for: Perception of the McGurk effect in people with one eye depends on whether the eye is removed during infancy or adulthood
Source: Front Neurosci. 2023 Oct 13;17:1217831. doi: 10.3389/fnins.2023.1217831 (PMC10603249; doi:10.3389/fnins.2023.1217831)

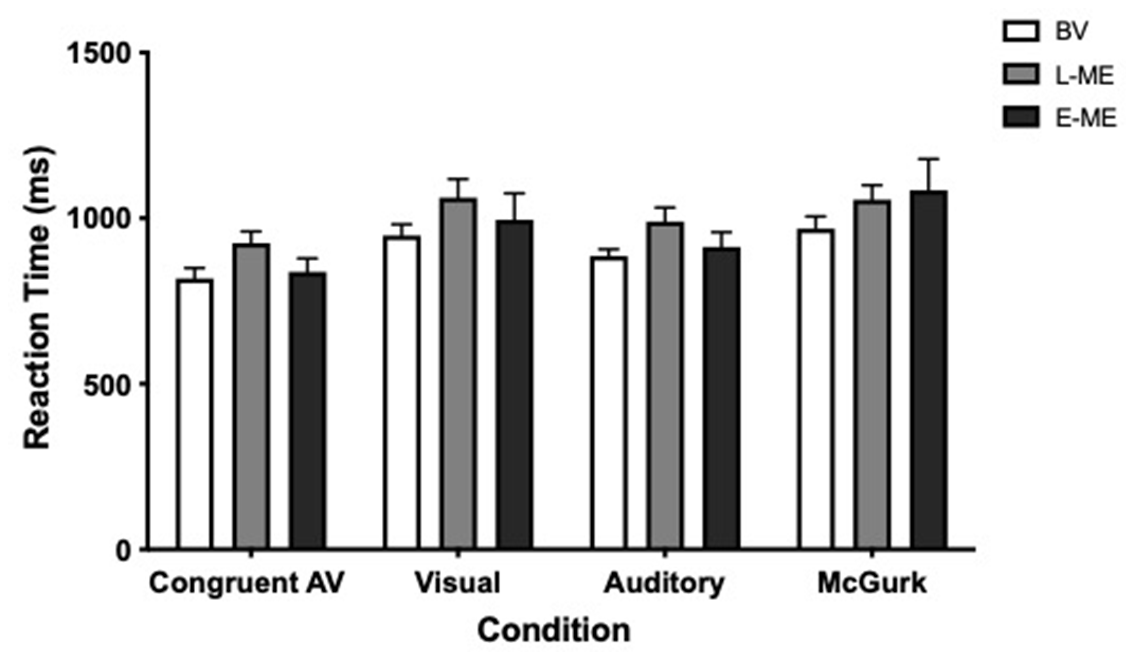

Supplement: Supplementary file 1 [file Image_1.tif]
