# Supplementary material for: Perception of the McGurk effect in people with one eye depends on whether the eye is removed during infancy or adulthood
Source: Front Neurosci. 2023 Oct 13;17:1217831. doi: 10.3389/fnins.2023.1217831 (PMC10603249; doi:10.3389/fnins.2023.1217831)
Supplement: Supplementary file 2 [file Presentation_1.pdf]

Perception of the McGurk Effect in people with one eye depends on whether the eye is removed during infancy or adulthood

Stefania S. Moro[1, 2], Faizaan A. Qureshi[1], and Jennifer K. E. Steeves[1, 2]

[1]Department of Psychology and Centre for Vision Research, York University, Toronto, Canada

[2]The Hospital for Sick Children, Toronto

1.0 Supplementary Materials

1.1 Comparing Online and In-Person Platforms

In order to determine whether there were differences in Accuracy, Reaction Time, or perception of the McGurk effect between online and in-person platforms we conducted a number of comparisons using repeated measures analysis of variance (ANOVA). Overall, there was no difference between the two platforms and as a result the two platforms were collapsed to yield one BV control data set (see supplementary materials for more details).

Reaction Time, Accuracy, and the Perception of the McGurk illusion were compared across the two Data Collection Platforms (Online and In-Person) for the BV control group. A Greenhouse-Geisser corrected, 2 × 4 repeated measures analysis of variance (ANOVA) comparing Platform (Online vs In-Person) and Condition (auditory only, visual only, congruent audiovisual, McGurk) revealed a non-significant interaction, $F(2.24, 62.58) = 1.36$, $p = 0.265$, $\eta_p^2 = 0.046$ and no main effect of Platform, $F(1, 28) = 0.239$, $p = 0.629$, $\eta_p^2 = 0.008$. There was a main effect of Condition, $F(2.24, 62.58) = 12.78$, $p < 0.001$, $\eta_p^2 = 0.313$. There was no difference in reaction time between the online and in-person platforms.

A 2 × 3 repeated measures analysis of variance (ANOVA) comparing Platform (Online vs In-Person) and Condition (auditory only, visual only, congruent audiovisual) was conducted. There was a significant interaction, $F(2, 56) = 6.63$, $p = 0.003$, $\eta_p^2 = 0.192$, main effect of Condition, $F(2, 56) = 42.76$, $p < 0.001$, $\eta_p^2 = 0.604$, and main effect of Platform, $F(1, 28) = 6.03$, $p = 0.021$, $\eta_p^2 = 0.177$. However, Bonferroni corrected post-hoc tests indicated that there was no difference between platforms at each of the different stimulus conditions.

A Greenhouse-Geisser corrected, 2 × 3 repeated measures analysis of variance (ANOVA) comparing Platform (Online vs In-Person) and McGurk Perception (perception of "Ba", "Ga", "Da" during incongruent audiovisual trials) revealed a non-significant interaction, $F(1.18, 33.04) = 0.103$, $p = 0.791$, $\eta_p^2 = 0.004$, and no main effect of Platform, $F(1, 28) = 2.14$, $p = 0.154$, $\eta_p^2 = 0.071$. There was a significant main effect of McGurk Condition, $F(1.18, 33.04) = 29.469$, $p < 0.001$, $\eta_p^2 = 0.456$.

1.2 Reaction Time

In order to determine whether there was a difference in auditory, visual, congruent audiovisual or McGurk perception performance with respect to Reaction Time between participant groups a Greenhouse-Geisser corrected, 3 × 4 repeated measures analysis of variance (ANOVA) comparing Participant Group (BV vs L-ME vs E-ME) and Condition (auditory only, visual only, congruent audiovisual, McGurk) was conducted. Results indicate a non-significant interaction, $F(4.77, 102.51) = 0.797$, $p = 0.549$, $\eta_p^2 = 0.036$ and no main effect of Participant Group, $F(2, 43) = 1.70$, $p = 0.195$, $\eta_p^2 = 0.073$. There was a main effect of Condition, $F(2.38, 102.51) = 26.987$, $p < 0.001$, $\eta_p^2 = 0.386$. There was no difference in Reaction Time between the E-ME group, L-ME group, and BV group. Figure 5 plots the Reaction Time for each for the BV, L-ME, and E-ME groups.

2.0 Figure Captions

Figure 5. Reaction Time for each for the BV (white), L-ME (grey), and E-ME (black) groups. There was no difference in Reaction Time between the groups. Error bars represent the standard error of the mean (SEM).
